# First steps in experimental cancer evolution

**DOI:** 10.1111/eva.12041

**Published:** 2013-01-03

**Authors:** Tiffany B Taylor, Louise J Johnson, Robert W Jackson, Michael A Brockhurst, Philip R Dash

**Affiliations:** 1 University of Reading, WhiteknightsReading, Berkshire; 2 Department of Biology, University of YorkYork, UK

**Keywords:** carcinogenesis, evolutionary trade-offs, kin competition, metastasis, resistance, resource competition, social evolution

## Abstract

Evolutionary processes play a central role in the development, progression and response to treatment of cancers. The current challenge facing researchers is to harness evolutionary theory to further our understanding of the clinical progression of cancers. Central to this endeavour will be the development of experimental systems and approaches by which theories of cancer evolution can be effectively tested. We argue here that the experimental evolution approach – whereby evolution is observed in real time and which has typically employed microorganisms – can be usefully applied to cancer. This approach allows us to disentangle the ecological causes of natural selection, identify the genetic basis of evolutionary changes and determine their repeatability. Cell cultures used in cancer research share many of the desirable traits that make microorganisms ideal for studying evolution. As such, experimental cancer evolution is feasible and likely to give great insight into the selective pressures driving the evolution of clinically destructive cancer traits. We highlight three areas of evolutionary theory with importance to cancer biology that are amenable to experimental evolution: drug resistance, social evolution and resource competition. Understanding the diversity, persistence and evolution of cancers is vital for treatment and drug development, and an experimental evolution approach could provide strategic directions and focus for future research.

## Introduction

Recently, there has been a shift in current thinking among cancer researchers that acknowledges the importance of evolution in understanding cancer progression within a host. This movement has been guided by an increasing body of seminal work (e.g. Vogelstein and Kinzler 1993; Leroi et al. 2003; Merlo et al. 2006; Attolini and Michor 2009; Sequist et al. 2011; Gerlinger et al. 2012; Greaves and Maley 2012) reinvigorating old ideas (Nowell 1976) regarding the evolution and ecology of cancers. Recent studies have detected rapid evolution and spatially structured genotypic and phenotypic diversity both within tumours and between primary and secondary tumours (Frumkin et al. 2008; Stoecklein et al. 2008; Sequist et al. 2011; Gerlinger et al. 2012). The consideration of evolutionary progression of cancers is therefore not just desirable it is essential. Cells become cancerous when mutations arise which increase their replication rate and survival advantage compared with neighbouring cells; subsequently, natural selection will act on these cells and ultimately ensure the proliferation of the mutant lineage (for reviews, see Crespi and Summers 2005; Merlo et al. 2006; Greaves and Maley 2012). Once cancerous traits have evolved, an elevated mutation rate and metastatic potential may facilitate diversification and persistence of the cancerous cells (Bielas et al. 2006). Moreover, aggressive drug treatment of cancers will unavoidably select for resistant lineages, which become increasingly difficult to treat. However, by understanding how the environment shapes the evolution of cancerous traits, we can begin to anticipate evolutionary trajectories and apply a more proactive treatment strategy (Cairns 1975; Aktipis et al. 2012; Yap et al. 2012).

There are a number of factors that will determine the fitness of cancerous cells within a host. The immediate microenvironment will present challenges for space and resources, and the extended microenvironment will provide further challenges in the form of vascularization and the realization of metastatic potential (that is the migration and colonization of cancer cells to secondary sites within the host). Although substantial effort has been conducted into understanding mechanistic consequences of cell–cell and cell–environment interactions, the evolutionary and ecological outcomes have not been explicitly tested. These have left fundamental questions regarding cancerous behaviours unanswered, such as: How do competitive interactions between cells determine disease progression? What are the fitness costs of drug resistance? How do the selective pressures between different environments effect disease progression (e.g. liquid versus solid cancers)? Answering these and other pressing questions in cancer biology will require carefully designed experiments that explicitly test competing evolutionary hypotheses of cancer progression, which are led by observational data from clinicians highlighting the medically important traits.

In this article, we propose that to answer some of the fundamental questions regarding the evolutionary progression of cancers, cancer biologists should adopt the powerful techniques of experimental evolution. Seminal experimental evolution studies have fundamentally changed our understanding of evolution in terms of adaptation (Lenski et al. 1991), diversification (Rainey and Travisano 1998), social evolution (Turner and Chao 1999; Velicer et al. 2000), evolutionary trade-offs and constraints (Novak et al. 2006; Blount et al. 2008), repeatability (Lenski et al. 1991; Lenski and Travisano 1994; Cooper et al. 2003; Woods et al. 2006; Barrick et al. 2009) and more besides; there is strong potential for similar advancements in cancer biology. Experimental evolution uses replicate populations of organisms with fast generation times (typically microorganisms) to study evolutionary processes in real time (for review see, Elena and Lenski 2003). The experimenter controls the environmental conditions under which evolution occurs and monitors the effect of specific selective pressures on traits of interest (Buckling et al. 2009). One major benefit of this system is that evolved and ancestral lines, or multiple evolved lines, can be competed against each other to measure fitness under defined ecological conditions, and the accumulation of mutations can be followed in a rigorously defined and time-directional manner over numerous generations.

Cancer cells can conveniently be grown *in vitro,* and these cancerous tissue cultures share many beneficial characteristics with microbial model systems used for experimental evolution studies (Table 1). A promising new area of research therefore suggests itself: experimental cancer evolution, which could provide new insights into disease progression and aid the strategic development of new drug therapies and treatment regimes.

**Table 1:** Features of microorganisms which make them an ideal model system for studying evolution experimentally (Elena and Lenski 2003) and parallels in cancer cells

Microorganisms	Cancer cells	Advantages for evolutionary experiments
Easy to propagate and enumerate	Immortal lines can be easily grown, and lines which have been used extensively in research for decades are well enumerated	Cells can be grown at low cost and in high volumes. Prior details of normal behaviour allow interesting mutants to be identified
Fast replication	Generation time of approximately 1 day	Allows experiments to conceivably run for many generations
Manipulable mutation rates	Elevated mutation rate compared to noncancerous cells	Facilitates variation by mutation within the population
Large populations exist in small spaces	Billions of cells can be grown in tissue culture flasks	Aids experimental replication
Stored easily and indefinitely in suspended animation	Cells can easily be frozen and revived	Enables comparisons between ancestral and evolved lineages; lineages can be catalogued and revived
Asexual reproduction	Cells divide mitotically	Clonality assists experimental replication
Easily manipulated experimental conditions and genetic composition of founding populations	Culture resources and environment are easily controlled	Allows identification of environmental and genetic influences on evolutionary processes; advancements in sequencing means genetic identification is easier and more cost-effective than ever before

Here, we discuss three general evolutionary problems that have the potential to dramatically influence the evolution of cancerous traits, but remain to be rigorously explored empirically: the evolution of drug resistance and associated costs, cooperation and conflict between cancerous and noncancerous cells, and resource competition as a driver for the evolution of metastasis. Although all three have already been successfully addressed using experimental evolution in microbes, cancers provide a new challenge to understand how predictions derived from simpler biological systems translate to a more complex one. Cancers have a comparably larger molecular ‘tool kit’ and a complex relationship within the ‘cell community’. Noncancerous cells are programmed for a multicellular lifestyle and will thus act altruistically for the benefit of the host, but cancer cells (which share the same signalling pathways) have the ability to manipulate these altruistic cells for selfish objectives. These factors have the potential to alter standard predictions made from unicellular models, but importantly will help to identify major issues which are likely to be key in the context of disease progression. To account for these differences between microbes and cancers (thus resulting in more accurate predictions regarding evolutionary trajectory), experimental evolution studies need to be conducted which explicitly test evolutionary theory within the context of cancer. A glossary is provided for definitions which might not be familiar to both fields (Box 1).

## Costs of resistance and trade-offs

The evolution of drug resistance is a process of adaptation by natural selection and has been well described by population genetics models (reviewed in, Levin et al. 2000; but see also Read and Huijben 2009). In particular, the relationship between mutation rate and the fitness effects of mutations is key. Genetic instability is a trait which is considered a hallmark behaviour (Hanahan and Weinberg 2011) of cancer. A high mutation rate is usually associated with a fitness cost, because most mutations are deleterious. However, a heterogeneous and frequently changing environment provides selection for phenotypes with elevated mutation rates, because increased genetic variation allows faster adaptation (Sniegowski et al. 1997). An accurate estimate of mutation rates of cancerous cells *in vivo* is yet to be determined; however, this information is key if we hope to better understand the predictability of resistance evolution. In particular, a newly developed approach by Wielgoss et al. (2011) uses whole genome sequencing combined with experimental evolution to provide a highly accurate measure of bacterial base-substitution rates – by sequencing several *E. coli* genomes after a 40 000 generation evolution experiment, they were able to directly infer the point mutation rate based accumulation of synonymous mutations. A similar approach could be adopted using cancer cells to try and identify why different cancers appear to vary in their mutation rate, and what impact mutagenic chemotherapy drugs have on baseline mutation rates (Loeb 2001).

Fitness effects of mutations are also crucial in determining their fate: resistance mutations are generally associated with a reduced fitness in the absence of the drug – this trade-off is termed the cost of resistance. Costs occur because resistance is typically achieved through alteration of the trait(s) targeted by the drug leading to impaired or lost function. If resistant lines carry a fitness cost but are still allowed to evolve in the presence of the drug, natural selection will act to counterbalance the cost while preserving the resistance, and as such resistant mutants will acquire new mutations which compensate for the fitness decline. These compensatory mutations are important in determining the probability of loss of resistance in a drug-free environment, because the fitness of mutants which have fixed compensatory mutations is conditional on the presence of the resistant mutations for which they compensate, thus in the absence of the resistance mutation, the compensatory mutations may carry a fitness cost, therefore reducing the likelihood of reversion (Schrag et al. 1997; Maisnier-Patin and Andersson 2004).

**Figure 1:**
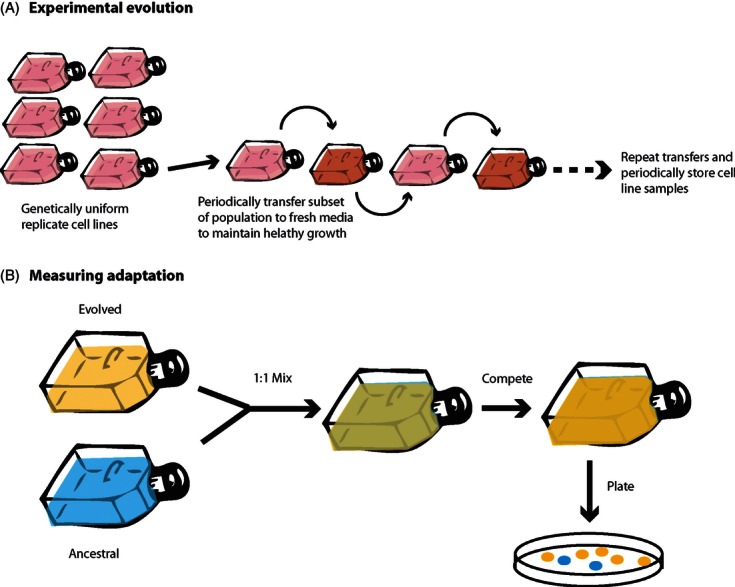
The simple competition experiment is one of the most powerful tools in experimental evolution. It allows ancestral and evolved populations to be directly competed to provide an estimate of relative fitness between populations under defined ecological conditions. Ancestral and evolved populations are grown separately, and then mixed at a 1:1 frequency. They are allowed to grow and compete, after which the frequency of each population is estimated by plating a subset of cells onto a hard media and counting each colony type (cells may need to be tagged to allow differentiation). After Elena and Lenski (2003) and Buckling et al. (2009).

Recent studies have seen rapid evolution of drug resistance in cancers which occur due to mutations in the epidermal growth factor receptors (EGFR), including non-small-cell lung cancer (NSCLC) and colorectal cancers (Kobayashi et al. 2005; Turke et al. 2010; Sequist et al. 2011; Diaz et al. 2012). EGFRs are essential for cell growth and development, and highly conserved across all animals (Bogdan and Klämbt 2001); for this reason, they are a common target site for cancer drugs, because by blocking the EGFRs, it is possible to slow cell growth. Sequist et al. (2011) found patients whose NSCLC had been treated with an EGFR blocker (tyrosine kinase inhibitor; TKI) evolved resistance within 12 months. To investigate the mechanism of resistance, genetic and histological testing was carried out on 37 patients with NSCLC treated with TKIs. It was discovered that all 37 samples had acquired new mutations related to the disruption of EGFR function, in addition to the original EGFR mutations which had triggered cancerous behaviour. This study clearly demonstrates that cancer cells which modified their EGFRs to disrupt binding of TKIs acquired resistance. However, changes to these receptors are likely to have large negative fitness effects on the cell, because they will be associated with less efficient cell growth, but the actual costs of these fitness effects remain to be explicitly measured. Competition experiments (Figure 1) could be used to quantify these costs and as such, improve the predictive power of the evolution of resistance to drugs which target EGFRs. Competition experiments involve directly competing ancestral and evolved (i.e. susceptible and resistant) populations of cells in order to estimate the relative fitness of each cell type. The likely large costs associated with changes to the EGFR are further exemplified in this study, because after some patients with resistant cancers stopped treatment with TKIs, the resistance mutations were lost, and their tumours once again became sensitive to treatment by either the same or a different EGFR inhibitor. Reversion may occur if the cost of the mutation conferring resistance is very high, but is unlikely if compensatory mutations have negated this cost. Therefore, in this specific study, the cost of resistance appears to be very high, and the effect of compensatory mutations, low – a promising observation in terms of managing drug resistance, and a measurable effect with *in vitro* testing.

Similarly, rapid evolved resistance to TKIs is seen in chronic myeloid leukaemia (CML) (Blagosklonny 2002; Shannon 2002; Shah et al. 2007). Resistance is acquired via point mutations to *BCR-ABL* (a gene responsible for constitutive tyrosine kinase activity) that cause structural changes which perturb binding of the drug (Gorre et al. 2001). Population genetics have been applied to clinical data to try and gain an insight into how TKIs exert its therapeutic effect in CML (Michor et al. 2005), finding that TKIs dramatically reduces the rate at which cancer progenitors are produced from the stem cells, but does not lead to a decline of stem cells themselves. In addition, they found the probability of evolving resistance increased with disease progression, as a consequence of increased stem cell abundance. This combined molecular and mathematical approach shows promising and informative direction for future development.

When resistance occurs, multiple drugs are commonly administered in the hope that bombarding the cells will mean full resistance is impossible (or at least improbable). However, the success of such an approach requires a detailed understanding of drug interactions: antagonistic interactions between drugs (i.e. drugs which inhibit each other's effects) will most likely lead to a low rate of evolution of multidrug resistance, because a mutation that confers resistance to one drug will be associated with only a marginal benefit; however, when interactions between drugs are synergistic (i.e. enhance each other's effects), resistance mutations will be associated with large benefits, and rapid multidrug resistance is expected (Hegreness et al. 2008; MacLean et al. 2010). There has been much research showing the importance of the microenvironment in determining cancer behaviour, and it is likely to be central in the context of evolution of drug resistance. By administering different drug strategies in the lab, which are known to be antagonistic or synergistic in their actions, under different environmental conditions (such as nutrient content, tissue type or genetic variability of tumour mass) we can test predictions regarding the evolution of drug resistance and gain a greater understanding as to what extent evolutionary trajectories are predictable.

In microbes, there has been a drive to understand the evolution of resistance due to the misuse of antibiotics leading to widespread antibiotic resistance. As such many techniques have been developed which measure the key components of the evolution of resistance, such as mutation rates, costs of resistance, compensatory evolution, multidrug resistance, and selection gradients. For instance, costs of resistance have been accurately measured using competition experiments (Figure 1) whereby the ancestral strain (which is susceptible to the antibiotic) and the resistant strain are grown together in the absence of the antibiotic. A subset of the population is then plated onto agar plates to allow growth of each strain type to be determined. Comparing the ratio of ancestral and resistant colonies enables fitness estimates to be made. This information allows one to determine the effect of antibiotic use under ideal conditions for microbial growth and predict consequences for *in vivo* use, enabling calculated risk assessment in antibiotic treatment. These experiments are all quick and simple to conduct and cheap to run – and similar experiments could be conducted using a cancer system.

In addition, a theoretical approach using adaptive walks allows predictions to be made regarding the likelihood of reversion of a resistance mutation to its susceptible ancestral genotype given a certain selection gradient and fitness landscape. Microbial data suggest that compensatory mutations are more likely than reversions, because there are several possible compensatory mutations for each resistance mutation (Poon et al. 2005). Moreover, compensatory mutations may be harmful in the absence of the original resistance mutation, further decreasing the chance of reversion (Schrag et al. 1997; Maisnier-Patin and Andersson 2004; Andersson 2006). Current research suggests that microbes cannot be relied on to lose resistance to an antibiotic if its use is discontinued (Andersson 2006; although see, Andersson and Hughes 2010), and therefore, we might predict that the rate of cancer cells losing resistance will depend on the type of drug used, the strength of selection and the degree of competition within the tissue (Komarova and Wodarz 2005; Szakacs et al. 2006). However, evidence from cancer research seems more promising. Several reversions to susceptible phenotypes were observed in one study (Sequist et al. 2011): but, how can we account for these apparent differences?

We suggest that this difference is due to disparities in drug action. Antibiotics can rapidly kill bacteria without destroying human cells because they exploit the differences between bacterial and eukaryotic cells. The differences between cancerous and healthy human cells are, by contrast, subtle, involving quantitative differences in gene expression (Zhang et al. 1997). Cancer treatment is therefore a more sensitive compromise between efficacy and toxic side effects, involving longer courses of treatment, typically several months. This means selection for drug resistance in cancer cells is weaker, but more prolonged, than selection for antibiotic resistance; mutations conferring resistance will be favoured, but any pleiotropic deleterious effects on fitness must be minimal. When bacteria are challenged with an antibiotic, however, selection pressures are strong, so mutations conferring resistance can fix even if they have considerably deleterious pleiotropic effects. Potential compensatory mutations are therefore more advantageous, and fix more rapidly. This hypothesis is testable using microbes and cancer cell lines.

## Social evolution: conflict and cooperation

The social environment – the behaviour of an organism's neighbours – can have a direct impact on fitness. These interactions are often thought of as negative: for instance, individuals that are competing for scarce resources will reduce each other's fitness. However, social interactions can also provide benefits if, for example, mutually secreted enzymes free up nutrients for neighbouring cells (Lee and Schneewind 2001; Nadell et al. 2010). Under these circumstances, the behaviour can be considered cooperative because the action benefits both the actor and the recipient (West et al. 2007). Cooperation can be split into two subgroups: mutualism, whereby both individuals contribute to a behaviour to which both gain benefit; and altruism, whereby an action benefits the recipient but comes at a cost to the actor. Altruism in particular posed a problem for evolutionary biology: how can a behaviour evolve which appears costly to the individual but beneficial to others (Maynard Smith and Szathmáry 1995; Hamilton 1996)? Hamilton (1964) explained the evolution and maintenance of altruistic behaviours through relatedness, whereby cooperative interactions can evolve if the benefit from such an interaction is most likely received by a kin member. This is because improving the fitness of a kin member helps to propagate the shared genes between the helper and the helped – thus an indirect benefit is gained. Mutual benefit cooperation on the other hand provides a direct benefit to all contributors, and therefore can evolve in the absence of high relatedness.

The evolution of multicellularity is considered one of the major evolutionary transitions in the history of life on earth (Maynard Smith and Szathmáry 1995). For multicellularity to evolve, cells are required to transition from unicellular individuals acting selfishly to proliferate their own survival, to being part of a group where survival is maximized between many cooperating individuals. For this to occur, competition between selfish entities must be repressed, and individual cell fitness aligned with group cell fitness (Leigh 1977), otherwise there will be a selective drive to defect from a multicellular lifestyle in favour of a solitary one (Frank 2003; Kümmerli et al. 2010). In our own bodies, individual somatic cells cooperate to maximize lifetime reproductive success, and thus transmission of the genes which are shared by every cell in our body. Cancer cells undergo this major transition in reverse, and abandon a cooperative existence to selfishly outgrow normal cells. At this point, the focused level of selection (in terms of understanding disease progression) shifts from the group to the individual cell (Klein 2003; Gardner and Grafen 2009).

However, this transition poses some interesting questions as to how these rogue cells interact with their social environment given that they possess the machinery to manipulate their competitors. Cell–cell interactions are vital in disease progression, resulting in complex heterotypic interactions where tumour cells interact with each other in addition to the normal stromal cells within the tissue microenvironment and *vice versa* (Weaver and Gilbert 2004; Axelrod et al. 2006). Noncancerous cells use signalling networks and mutually secreted enzymes in normal cell growth; however, cancer cells use these same pathways and signals for abnormal cell growth. Cancer cells are known to interact with each other and with cells from the tumour microenvironment – recruiting noncancerous cells to facilitate proliferation (Hanahan and Coussens 2012), and recent research has noted the conceptual similarities between cancer and bacteria in terms of social behaviours (for review see Ben-Jacob et al. 2012). There is evidence that certain hallmark behaviours (Hanahan and Weinberg 2011) of cancer cells require the excretion of products (sharable resources) which will be beneficial to other cancer cells within the vicinity (Stetler-Stevenson et al. 1993; Coussens et al. 2002; Egeblad and Werb 2002): this is cooperative behaviour. Cooperative behaviours are intrinsically involved in several steps of tumour progression, such as angiogenesis, self-sufficiency in growth signals and tissue invasion (Table 2). This leads to a scenario of both cooperation and conflict, whereby cancer cells are regarded as cooperators in terms of the disease, but create conflict (i.e. behave as cheats) in the eyes of the host.

**Table 2:** Examples of ‘mass action cooperation’ (Heckathorn 1996) in cancers, where mutualisms form between different clones and cells within the microenvironment

Behaviour	Cooperative characteristics
Angiogenesis	Tumours require nutrients and oxygen to grow. Therefore, they must recruit new blood vessels into the area (neoangiogenesis) by secreting vascular endothelial growth factor (VEGF). The recruitment of blood vessels to an area not only benefits the secreting cells but also any cells within the local vicinity. Therefore, VEGF can be thought of as a communal product, the production of which is likely to carry an energetic cost to the producer, and a growth benefit to any recipients. Evidence for this behaviour has already been shown in cancer–stromal–cell interactions, and there is growing evidence that it may also be important between cancer cells (Kalas et al. 2005).
Self-sufficiency in growth signals	Cancer cells produce many growth factors (such as VEGF, PDGF and TGF-β) (Mueller and Fusenig 2004) that induce stromal reactions for angiogenesis and inflammation, and activate other stromal cells, such as fibroblasts, to secrete other growth factors (GFs) and proteases (de Jong et al. 1998; Klein 2003; Weaver and Gilbert 2004). Although not systematically tested, there is some evidence that cancer cells secreting GFs are frequently adjacent to cells which express GF receptors, and therefore have the potential to respond to GF signals (de Jong et al. 1998; Royuela et al. 2004). This suggests that cancer cells are able to share GFs between each other, and also recruit GFs from noncancerous cells, which will aid tumour growth.
Tissue invasion	Cancer cells interact with stromal cells to stabilize the tumour microenvironment. Normally, tissue cells remain confined to their territory because they respond to signals from neighbouring cells, and the extra cellular matrix (ECM). Any cells which become detached receive apoptotic signals from the invaded tissues, and as such are eliminated. Malignant tumour cells effectively ignore these signals, and so are able to migrate beyond the defined boundaries of the tissue (Liotta and Kohn 2001). Stromelysin-3 secreted by fibroblasts peripheral to the tumour is known to reduce the death rate of cancer cells invading adjacent connective tissues. In addition, proteases from nearby stromal and cancer cells are known to contribute to neoplastic progression by degrading ECM, aiding in cell proliferation, tissue invasion and metastasis (Tlsty 2001).

The tensions between cooperation and conflict over shared resources has been extensively researched in microbial systems, and some seminal papers have fundamentally changed our view of the social world microbes live in (Chao and Levin 1981; Turner and Chao 1999; Strassman et al. 2000; Velicer et al. 2000; Queller et al. 2003; Fiegna et al. 2006). Bacteria produce numerous extracellular molecules, such as tissue degrading enzymes, iron-scavenging siderophores and sticky polymers to protect surface growing bacteria (biofilms), which are individually costly but benefit the group as a whole (West et al. 2007). Using techniques from experimental evolution on microbial systems has enabled us to explore theories regarding the evolution of cooperative behaviours, and gain a better understanding for how they might be maintained within a population.

Evidence suggests cooperation will be maintained through indirect and direct fitness benefits (Griffin and West 2002). Indirect fitness benefits require high relatedness within the population, ensuring cooperative behaviours benefit local kin members. Direct fitness benefits can occur if individuals have a shared selfish interest in cooperating, for example, in the case of cross-feeding (where the waste product of one species is an energy source for another; Bull and Harcombe 2009). Moreover, it has been successfully established, using microbes, that social behaviours often involve frequency-dependent selection (Queller 1984). This is because a ‘cheating behaviour’, that is letting those around you contribute to a public good so you don't have to, is only a successful strategy if it is rare. This is likely to be important in the context of cancers because these factors will limit the size at which tumours can successfully function. The influence of such factors is easily addressed using competition assays (Figure) which vary in the initial frequencies of altruists and cheats (Ross-Gillespie et al. 2007).

Bringing social evolution research into the context of cancer will give new insights into the levels of selection and transitions to multicellularity. The experiments can start simple: spheroids (artificial masses of cancer cells), which are made up from cells of varying genetic diversity, are exposed to a range of environments which alter the competitiveness between cells (e.g. varying nutrient conditions). Here, we can look to see whether the predictions made by social evolution theory are fulfilled, and build on this knowledge with more complex experiments. Due to the easy manipulation of spheroid diversity, it gives the potential for cancer to make an elegant system on which some important questions can be answered: How does relatedness influence relative competition between cells? Is competition beneficial (because it encourages cooperative behaviours and metastasis) or disadvantageous (because cells have less individual resources) for tumour growth?

## Resource competition: dispersal and metastasis

Metastasis in cancer is conceptually equivalent to dispersal in ecology. As disease progresses, cells detach from the primary tumour and circulate in the bloodstream, where some go on to colonize new tissues and establish metastatic tumours (Friedl and Wolf 2003). Likewise, dispersal is the relocation of individuals from a natal site, a behaviour which provides the opportunity for population expansion via colonization events. It is therefore essential to apply the well-established evolutionary theories of dispersal to understand metastasis.

Dispersal is a risky strategy: a dispersing individual may die, settle in an unsuitable habitat or find itself in competition with locally adapted rivals, and for many organisms, the probability that dispersal will pay off is extremely low. Yet dispersal is ubiquitous across the spectrum of life: from microbes, to plants, to birds. To understand how dispersal strategies evolve, one must understand the costs and benefits which will determine when and how dispersal is favoured, and the ecological subtleties which shape the behaviour. Fundamentally, the benefit of dispersal is that it allows an individual to escape local competition (providing an indirect fitness benefit) and gain access to resources (providing a direct fitness benefit) (Bowler and Benton 2005; Wei et al. 2011). A crucial breakthrough was the recognition that dispersal is often a social trait such that organisms might pay high costs for dispersal if they are surrounded by kin. When individuals within the population are genetically similar (termed related), the overall benefit of dispersal is maintained, even when individual costs of dispersing are high (Hamilton and May 1977; Comins et al. 1980; Taylor and Frank 1996; Gandon and Michalakis 1999; Taylor and Buckling 2010). This is because the inclusive fitness of an individual is determined not only by how many genes it directly passes onto the next generation but also by how many genes shared with related individuals are also passed on (termed indirect fitness). This social consideration in terms of dispersal supports the paradoxical observation of how dispersal might evolve even when most dispersing individuals themselves die: a dispersing individual can increase the chances that related individuals, who forego dispersal to compete for resources, will benefit from decreased competition and consequently the disperser will benefit indirectly.

Limited dispersal will increase relatedness (favouring the evolution of altruistic traits), but increase local competition among relatives (disfavouring altruism). Theoretically, in the simplest scenario, these two factors exactly cancel each other out, such that the level of dispersal has no effect of the evolution of altruism (Taylor 1992). However, the consideration of more complex scenarios provides mechanisms for ways in which limited dispersal can still favour altruism (Lehmann and Keller 2006; Alizon and Taylor 2008). One such mechanism which can provide a solution to the maintenance of social traits with dispersal is the process of budding dispersal. Experimental evolution has shown that this trade-off between limited dispersal and increased local competition can be circumvented when cooperators disperse in small aggregated groups (Kümmerli et al. 2009), thus keeping relatedness high and allowing the colonization of new patches where competition is low. There is evidence that cancer cells do not only migrate individually, but also collectively, in a behaviour comparative to budding dispersal (Friedl and Wolf 2003). This behaviour increases tumour invasion efficiency and survival probability through the maintenance of social behaviours, for example: the larger cell mass maintains high endocrine concentrations, protects inner cells from risks encountered during dispersal, and also promotes invasion of cells which are less mobile. It is yet to be determined how common metastatic secondary tumours are established via collective-cell movement, but evolutionary theory would predict that the disruption of group migration would decrease metastatic formation under this scenario.

One theoretical finding of particular relevance is that population processes and the evolution of dispersal are highly interdependent. Specifically, demographic features of the population will determine dispersal rate, and dispersal rate will alter population demography (Ronce 2007). To understand the maintenance and stability of dispersal strategies, it is vital to consider feedbacks between evolutionary and ecological processes associated with dispersal. In cancers, for example, the increasing cell density in a growing tumour will alter the local environment (especially the intratumour microenvironment), rapidly reducing nutrient levels and oxygen availability; this, in turn, will feedback into the population demography – increasing relative competition between cells for a dwindling pool of resources. This competition may change relative selective pressures: decreasing the benefit gained from associating with nearby cells, and increasing the benefit of dispersing, which will provide the opportunity to escape competition and potentially colonize new empty patches.

Metastatic cancers are the most aggressive and have the poorest outcomes in terms of patient survival (Liotta et al. 1991). However, only a vanishingly small proportion of metastatic cells establish a new tumour (Butler et al. 2000), and metastatic cells are at a significant disadvantage in competition for space and resources (Chen et al. 2011). The problem of explaining how metastasis evolves despite high apparent costs was identified by Bernards and Weinberg (2002): ‘(T)here is no reason to think that a metastatic phenotype enables cells to proliferate more effectively within the primary representation in the overall tumour-cell population’. In other words, metastasis should only exist as a rare trait, overwhelmingly dominated by static phenotypes which do not pay the cost of dispersal. Unfortunately, this does not describe the observed patterns of metastatic progression (Liotta and Kohn 2003), with an estimated 10^6^–10^7^ cells emigrating daily from a developed neoplasm (Butler et al. 2000).

Dispersal ecology theory was recently applied to this problem, and metabolic rate, determined by resource heterogeneity within primary tumours, was considered a selective agent for high cell motility (Anderson et al. 2006; Chen et al. 2011; Aktipis et al. 2012). The microenvironment surrounding a growing neoplasm can quickly change over small distances, becoming hypoxic (Harris 2002; Brurberg et al. 2003, 2005; Vaupel and Harrison 2004; Cárdenas-Navia et al. 2008) due to poorly regulated angiogenesis, changes in vascular architecture, or temporary obstruction or interruption of blood flow by neoplastic cells (Boucher and Jain 1992; Araujo and McElwain 2004; Vaupel and Harrison 2004). The effect of spatial and temporal resource availability was modelled mathematically with the conclusion that resource heterogeneity selects for cell motility, and cell dispersal is an evolutionary consequence of that selection (Chen et al. 2011).

Insights from experimental evolution using microbial model systems show that kin competition can indeed drive the evolution of dispersal behaviour. Taylor and Buckling (2010) found that the benefit of dispersal was much higher in clonal (highly related, or kin based) bacterial populations, and these clonal populations were more likely to disperse under very high resource-dependent costs than mixed dispersal strategy populations. In addition, Wei et al. (2011) found bacterial motility provided large benefits to a bacterial population by allowing individuals to move away from each other and thereby obtain a greater share of resources in physically structured environments. These types of studies provide a framework to develop similar experiments in a cancer cell system (Box 2), which will provide vital data on basic questions concerning metastatic behaviour, for example: Is metastasis a heritable trait? To what extent does environment determine metastatic behaviour over genetics? and What role does the cost of dispersal play in determining the success of new tissue invasion, and how does the social environment alter these costs?

Crucially, cancer cells do differ from microbes, and their own natural history must be considered. Even including transmission *in utero*, there are extremely few known instances in which a human cancer has dispersed outside its original host, and no known outbreaks of repeated host-to-host transmission (Dingli and Nowak 2006). Of the three transmissible tumours known from other mammals, only canine transmissible venereal tumour (CTVT) has spread through a typical host population. Devil facial tumour disease (DFTD) infects Tasmanian devils, a critically endangered species with low genetic diversity, and transmissible hamster sarcoma spread via a vector through laboratory animals which shared sufficient genetic identity (Banfield et al. 1965).

We posit that in the long run, a cancerous cell line is doomed, because they have not evolved a long-term strategy to promote vertical or horizontal transmission. Of course, evolution has no foresight, so this ultimate futility does not in itself affect the progress of disease. However, selection for colonization ability can only occur at colonization events; even where selection is very strong, it must also be iterative to build up complex adaptations. Cancers undergo relatively few colonization/dispersal cycles, and consequently cannot build up the intricate adaptations for dispersal and establishment found in independent organisms; instead, they may crudely redeploy existing motility pathways, such as those used in development and wound healing. This may result in maladaptive or suboptimal behaviours that could be exploited in cancer treatment.

## Concluding remarks

In this article we have discussed how methods from experimental evolution can be applied to help understand the evolution of cancers. Considering cancers from an ecological and evolutionary perspective should lead to innovative approaches to disease control and drug treatment. There are many lessons to be learnt from evolutionary theory, and a cancer tissue model system shares many of the advantages of a microbial model system, providing great potential for addressing evolutionary questions regarding disease progression in a biologically relevant system. In cancers, this model system has the advantage of direct applicability to the disease model. In particular, understanding the repeatability of evolutionary processes would have significant clinical applications for cancer biology, as currently, cancer types are often treated as distinct from each other, requiring independent avenues of research in order to understand their differences. However, by focusing on the similarities between cancers rather than the differences, universal treatment strategies may be identified.

We must, however, consider the limitations of experimental evolution and despite its proven success in microbial models – the methods have also received criticism (Buckling et al. 2009), namely concerning its realism to more natural settings. The laboratory is not a natural environment, and therefore some argue the results are not applicable to the ‘real-world’. However, during the course of an experimental study, an organism will adapt to its laboratory environment (in fact, many commonly used cancer cell lineages have been maintained in laboratory conditions already for many generations). Therefore, these organisms will be adapted to the environment in which they are assayed – the alternative is to take organisms out of the environment to which they are adapted and measure them under laboratory conditions. This will likely mask the effects of the selective agent being considered, as expression of the trait of interest may be modified by unfamiliar surroundings. Furthermore, the approach has been criticized for being too simplistic in comparison to real-world complexity. However, the simplicity of design is the exact benefit of this method – using a simple model system captures the influence of certain identified selective factors on a trait of interest – thus improving overall generality of the results.

Identifying mechanisms that directly influence the evolutionary progression of a disease requires in depth understanding of the genetic, behavioural and physiological components within a phenotypic context. The first step in this process is the development of evolutionary theory in a mathematical model to identify and quantify the selective factors which determine evolutionary processes. Evolutionary models are now being used to explore the evolutionary progression of cancers, and opening the door for communication between disciplines in the process (Nunney 2003; Anderson et al. 2006; Chen et al. 2011). However, without empirical synergy, the true applicability of these models is difficult to determine. Empirical systems bridge the gap from theory to clinical reality, which enables the translation of ideas and evolutionary risk analysis of drug treatment and disease progression.

The three examples of relevant evolutionary questions discussed above are by no means an exhaustive list. The first step for these experimental evolution studies should be to identify the population dynamics which will influence evolutionary processes, such as measurement of mutation rates, fitness effects of mutations, generation times, population structure, the frequency of selective sweeps and the selective effects of drug therapies (Merlo et al. 2006). The second step should be to identify the ecological effects different environmental conditions have on such processes. The third step should be to identify the genetic mechanisms underlying the evolution of disease-related behavioural changes. Finally, we should be able to apply such knowledge to patient-specific cases, with the hope of pre-empting progression of the disease with personalized treatment strategies.

**Glossary**

**Table:** 

Adaptive walks	Sequences of beneficial mutations
Altruistic	An action directed towards another individual which results in a cost to the helper and a benefit to the helped
Angiogenesis	The physiological recruit of new blood vessels
Compensatory mutations	Mutations which offset the negative fitness effects imposed by another mutation
Cooperative	An action which benefits both the helper and any helped
Demographic features	Characteristic features of a population
Direct fitness	An individual's own genetic contribution to the next generation
Epidermal growth factor receptors	Surface growth factor receptors
Fitness landscape	A multidimensional space where an artificial landscape comprised peaks and valleys represents a genotype or phenotype fitness value
Hallmark behaviours	Common traits of cancer cells
Hypoxic	Oxygen depleted environment
Inclusive fitness	The sum of an individual's direct and indirect fitness
Indirect fitness	The genetic contribution to the next generation gained from the reproduction of relatives
Kin member	Genetically related individual
Metastatic (metastasis)	Secondary tumours caused by the migration of cells from the primary tumour to other tissues within the body
Mutualism	Ecological relationship beneficial to both partners
Neoplasm	An abnormal tissue mass
Phenotype	Observable characteristics of an individual resulting from the interaction of its genotype with the environment
Reversion	Back mutation of a point mutation to its ancestral state
Relatedness	The level of consanguinity between two given individuals
Selection gradient	The slope of a regression of fitness on trait value
Somatic cells	Cells which make up the tissues of the body (i.e. not the germ cells)
Stromal	The supporting tissue of an organ
Tyrosine kinase inhibitor (TKI)	A drug that interferes with cell communication and growth and may prevent tumour growth

**A Thought Experiment**

Using *in vitro* techniques to understand how resource competition between cells can drive metastasis in cancers – an experimental evolution approach

Metastasis – the progressive spread of malignant cells away from its origin to colonise new tissues – is the most deadly aspect of cancer, and therefore, understanding the processes which encourage cell movement is of integral interest to the field. From the cancer's perspective, metastasis offers the opportunity to escape deteriorated patches and colonise unexploited, healthy tissue. Dispersal theory has been applied to cancer research, giving evolutionary explanations for trends in cell motility via a Darwinian fitness approach. Such theory predicts that increased competition between cells, via factors such as resource depletion, should promote dispersal and thus encourage metastasis.

**Prediction:** More motile cells will be fitter when competition between cells is greater

**Methods:**

1. Set up replicate cell lines of non-metastatic spheroids under two media conditions: (i) high nutrient media and (ii) low nutrient media.
2. Adhere spheroids to plates to enable motility to be expressed
3. Transfer a subset of the population to new media (within the same media treatment conditions) periodically to maintain population growth. Continue for a number of generations, until a phenotypic difference between cell lines is detected.
4. Measure difference in motility between cell lines, and compete evolved lines with ancestral lines under different nutrient conditions to see whether evolved lines have a fitness advantage.

**Predicted Results**

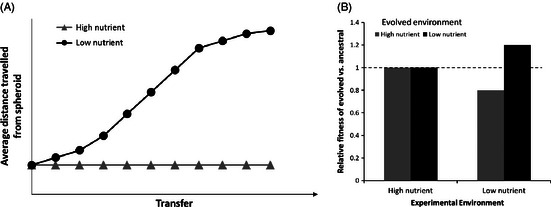
 A qualitative prediction showing: (a) cells which are evolved in a low-nutrient environment will become more motile than those evolved in a high-nutrient environment over time, and (b) when evolved and ancestral cell lines are competed in high- and low-nutrient conditions, the relative fitness (proportion of the evolved compared to the ancestral cells) of cell lines evolved in a low-nutrient environment will be higher than those evolved in high nutrients as they are able to escape competition and access more resources than those evolved in high nutrient. Under high-nutrient conditions, there is no benefit to dispersal and therefore cell lines are equally fit.
